# Simultaneous Low- and High-Grade Primary Leiomyosarcomas in Two Separate Organs in a Thirty-Year Survivor of Hereditary Retinoblastoma

**DOI:** 10.1155/2015/968567

**Published:** 2015-12-30

**Authors:** Katherine Moore, Ossama Tawfik, Allyson Hays, Isaac Opole

**Affiliations:** ^1^Department of Internal Medicine, The University of Kansas Medical Center, Kansas City, KS 66160, USA; ^2^Department of Pathology and Laboratory Medicine, The University of Kansas Medical Center, Kansas City, KS 66160, USA; ^3^Department of Hematology/Oncology, Children's Mercy Hospital, Kansas City, MO 64108, USA; ^4^School of Medicine, University of Missouri-Kansas City, Kansas City, MO 64108, USA

## Abstract

Patients with hereditary retinoblastoma have a heightened risk of developing subsequent bone and soft tissue sarcomas, exacerbated by radiation and alkylating chemotherapy. Secondary leiomyosarcomas are rare. A 29-year-old patient with bilateral retinoblastoma history presented with concurrent, independent malignancies found to be Rb-suppressed uterine and Rb-positive bladder leiomyosarcomas. She had undergone both ifosfamide chemotherapy and radiation therapy but demonstrated rare simultaneous primary leiomyosarcomas not previously described in association with ifosfamide. This rare case underlines the protean genetic predisposition to malignancy in this patient population, heightening awareness of lifelong malignancy potential, and the necessity for continuous periodic screening.

## 1. Introduction

Although a rare neoplasm, retinoblastoma is the most common eye tumor in children. It is frequently caused by mutations in the Rb-1 tumor suppressor gene located at band q14 of chromosome 13 and is hereditary in 30–40% of patients. When hereditary, the disease is more likely to be bilateral and multicentric.

Patients with a history of hereditary retinoblastoma are more likely to develop soft tissue sarcomas, bone sarcomas, and malignant melanomas [1, 2]. Leiomyosarcomas have rarely been reported but when present tend to occur in the radiation field adjacent to the orbit and maxilla [2]. Only a few visceral leiomyosarcomas have been reported [3–5], and the simultaneous development of two independent visceral leiomyosarcomas as secondary malignancies after retinoblastoma has not been described. We report a 29-year-old female patient with history of bilateral retinoblastoma diagnosed in infancy, who subsequently developed two distinct simultaneous leiomyosarcomas of the urinary bladder and uterus, in addition to osteosarcoma of the femur at age 12 years.

## 2. Case

A 29-year-old female presented with nausea, vomiting, diarrhea, and abdominal pain. Her past medical history was significant for bilateral retinoblastoma. Her right eye was enucleated at four months, and one year later retinoblastoma was identified in the contralateral eye. She underwent external beam radiation and chemotherapy with vincristine and cyclophosphamide for three years. At age 12 she developed osteosarcoma of the right femur for which she received adriamycin, ifosphamide, and methotrexate for one year, with subsequent limb salvage surgery with allograft. At age 29, she presented to our hospital and underwent imaging that demonstrated a vascular mass in the posterior bladder with incidental findings of fibroid uterus and right ovarian cyst. Exploratory laparoscopy, cystoscopy with biopsy, and pelvic washing revealed a bladder neoplasm consistent with high-grade leiomyosarcoma. Gynecologic evaluation showed an endometrial polyp with simple hyperplasia without atypia in the curettage specimen and rare atypical malignant cells suggestive of either an adenocarcinoma or a spindle cell neoplasm. Further radiological surveys of the chest, abdomen, and pelvis did not reveal any metastatic disease, although a bone scan showed a moderate focus of activity in the right proximal humerus; this was subsequently consistent with enchondroma.

The patient underwent a radical cystectomy with ileal conduit and simple hysterectomy. The cystectomy specimen showed a tan, partially necrotic papillary tumor with edematous and focally hemorrhagic surrounding mucosa. The tumor did not extend throughout the bladder wall, and there was no connection between the posterior wall of the bladder and the anterior aspect of the uterus. The hysterectomy specimen revealed multiple leiomyomatous lesions in the anterior and posterior uterine walls and cervix that varied from small microscopic lesions to up to 3.2 cm in largest diameter.

**Figure 1:**
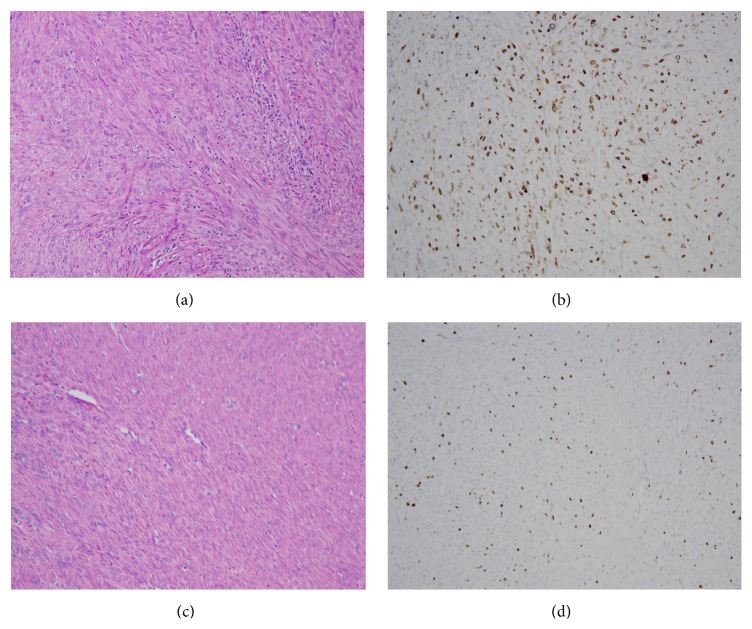
(a) Bladder leiomyosarcoma, H/E 200x: sheets of spindle shaped cells with disordered architecture and multiple mitotic figures. (b) Bladder leiomyosarcoma, MIB-1 immunohistochemistry, 200x: extensive MIB-1 positive cells showing high proliferative activity. Cells were also Rb, SMA, desmin, and vimentin positive, while negative results were obtained with S-100, melanin cocktail, CD117, and CD10. (c) Uterine leiomyosarcoma, H/E 200x: well-differentiated extensive low-grade leiomyomatous architecture with few mitotic figures. (d) Uterine leiomyosarcoma, MIB-1 immunohistochemistry, 200x: lower MIB-1 proliferative activity showing low-grade malignancy positive for ER and PR and negative for Rb and p53.

Histopathological analysis demonstrated two distinct leiomyosarcomas: one arising from the urinary bladder, the other from the uterus. These were established to be unrelated, representing two independent primary sarcomas. The bladder leiomyosarcoma was high-grade with mitotic figures averaging 80/10 high power fields. The tumor was positive for Rb, p53 (1-2%) and negative for estrogen receptor (ER) and progesterone receptor (PR) and had a higher MIB-1 (Ki-67) proliferation marker count (50%) (Figures 1(a) and 1(b)). In contrast, the uterine leiomyosarcoma was low-grade, well differentiated with a lower mitotic count (10/10 high power fields). It arose in a background of diffuse leiomyomatosis that extensively involved the myometrium. This leiomyosarcoma was positive for ER and PR and negative for Rb marker MIB-1 count which confirmed the increased but lower proliferative activity, as compared to the bladder leiomyosarcoma (10–15%) (Figures 1(c) and 1(d)). Additional confirmatory markers for leiomyosarcoma included vimentin (+), smooth muscle actin (+), desmin (−), S-100 (−), CD117 (−), melanoma marker (−), and CD10 (−).

All other specimens including pelvic lymph nodes received were without malignancy, and all resection margins were free of tumor. The patient has undergone follow-up for seven years and remains cancer-free.

## 3. Discussion

The Rb gene acts as a tumor suppressor gene in all cells of the body as it does in the cells of the immature retina. When Rb is mutated in a germline fashion, patients with hereditary retinoblastoma are at high risk of developing a second nonocular malignancy, with an estimated cumulative incidence of approximately 1% for each subsequent year of life [1]. The use of radiation therapy in hereditary patients with retinoblastoma increases the incidence of new cancers at 50 years of age to 38%, compared with 21% in nonhereditary RB survivors [4]. The most common second malignancy is bone sarcomas, specifically osteosarcoma which tends to occur within 25 years of RB diagnosis [4]. The role of alkylating chemotherapy and radiation therapy in hereditary retinoblastoma survivors appears to be additive [5].

Soft tissue sarcomas are another group of secondary malignancies that have been reported [1–3, 6–12]. They most often develop in the head and neck [7] and are often attributed to the destabilizing DNA effects of external beam radiation. There are only a few case reports of malignancies developing in visceral organs or in the distal extremities [1–3, 8]. In a recent large retrospective study of retinoblastoma survivors, a significant number who developed soft tissue sarcoma also had another cancer preceding their soft tissue sarcoma, and osteosarcoma was the most common. However, leiomyosarcoma was the most common histologic subtype of soft tissue sarcomas presenting as a secondary malignancy [6]. The patient described in this report seems to follow this pattern. External beam radiation increases the risk of second malignancy and shows a radiation dose-response relationship for all sarcomas [9], but it has been observed that leiomyosarcomas are diagnosed more frequently outside the field of radiation than in it [6], giving credence to a theory of genetic predisposition rather than a simple mutagenic effect of radiation.

Leiomyosarcoma of the urinary bladder represents less than 1% of all bladder cancers [10]. There have been isolated case reports describing bladder leiomyosarcoma as a secondary malignancy [1, 8, 10–13] and most are associated with the use of cyclophosphamide as a chemotherapeutic agent for treatment of the retinoblastoma due to alkylating method of DNA damage [10–13]. However, there is one report of a patient who developed urinary bladder leiomyosarcoma without cyclophosphamide treatment [8] while there are multiple cases reported of bladder leiomyosarcoma after cyclophosphamide treatment for malignancies and diseases other than retinoblastoma [14]. Ifosphamide rather than cyclophosphamide was used in our patient and although the two agents share chemical structure and mechanism of action, there have been no prior reports suggesting increased risk of bladder leiomyosarcoma after ifosphamide treatment.

Uterine leiomyosarcoma as a secondary malignancy after hereditary retinoblastoma has also been evidenced in a few retrospective studies, although no case reports have been specifically described. Loss of heterozygosity of Rb gene has been reported in uterine leiomyosarcomas [15] which is consistent with our case. This report describes a unique case of a young lady who survived bilateral retinoblastoma in infancy and subsequently developed osteosarcoma, followed later by two independent leiomyosarcomas of the urinary bladder and uterus. This rare case demonstrates the high genetic predisposition to malignancy in this patient population, which may be compounded by additional factors such as chemotherapy and radiation [4, 15, 16]. The extent to which her previous treatment regimens may have contributed to subsequent malignancy is unknown. However, her case emphasizes the importance of life-long awareness in survivors of hereditary retinoblastoma due to the heightened risk for multiple malignancies.
